# Editorial: Reviews in neurogenetics

**DOI:** 10.3389/fneur.2026.1979264

**Published:** 2026-09-14

**Authors:** Lucia F. Cardo

**Affiliations:** Cardiff University, Cardiff, United Kingdom

**Keywords:** Ataxia, cerebrotendinous xanthomatosis (CTX), Charcot-Marie-Tooth (CMT), CYP27A1 gene, PRUNE1 syndrome, RNA-based therapeutics

In this editorial we summarize the contributing articles to the Research Topic “*Reviews in neurogenetics*” of the journal Frontiers in Neurology. The articles contribute to advances in Neurogenetic disorders diagnosis and therapeutic avenues of high prevalent disorders as Dementia or Ataxia, and highlight the need to use similar approaches for less represented disorders as Charcot-Marie-Tooth, cerebrotendinous xanthomatosis or PRUNE1 syndrome.

## Clinical trials in Charcot-Marie-Tooth Disorders: a retrospective and preclinical assessment

Nair et al. present an interesting retrospective of Clinical trials in Charcot-Marie-Tooth Disorders between 1999 and 2022. Their work provides evidence of substantial growth in trials focusing on procedural and targeted gene therapy approaches. Majority of the trials were carried out in Academic settings (91%) while a minority was led by pharmaceutical companies (9%). Eighty-six percent of the trials were classified as therapeutic, and between 28% and 38% (depending on setting) were controlled, randomized and double-blinded. Thirty-two countries participated with the United States accounting for 26% (75/286), 36% of the Trials resulted in an Academic publication.

In this retrospective, the Authors observed a trend of rapid expansion of procedural trials including early investigational drugs utilizing viral and non-viral gene therapies. Promisingly, there is evidence of substantial growth in trials focusing on procedural and targeted gene therapy approaches.

## Neuroimaging in PRUNE1 syndrome: a mini-review of the literature

Scorrano et al. reviewed PRUNE-1 syndrome, a rare neurodevelopmental disorder presenting hypotonia, microcephaly, and variable cerebral anomalies amongst other features. The review focuses on clinical and radiological spectrum based on the pathogenic variants described to date. They explored the neuroradiological findings that, in combination with clinical phenotypes and genetic data, will allow to best characterize children affected. Their findings have diagnostic and potential prognostic implications.

## Decoding the genetic blueprints of neurological disorders: disease mechanisms and breakthrough gene therapies

Saeed et al. performed a comprehensive review focused on the genetic foundations of various neurological conditions from Cerebellar Ataxias, Paraplegias, Dementias to Huntington's disease or MSA. Of particular interest is their exploration of the potential of RNA-based therapeutics, with gene therapies standing at the forefront of precision treatment approaches. Through its comprehensive approach, the Authors aim to lay the groundwork for future research and clinical interventions. Their goal is that progress in the field progresses toward more effective treatments and better quality of life for individuals affected by these neurological conditions. Their hope moving forward is that the combination of genetic studies, emerging therapeutic strategies, and neuroimaging techniques, could address more effectively the understanding and treatment of these complex conditions.

## Clinical and genetic analysis of a family with cerebrotendinous xanthomatosis

In this study, Guoliang et al. analyzed the clinical and genetic causes of cerebrotendinous xanthomatosis (CTX) in a Chinese family. CTX is an autosomal recessive lipid storage disorder that results from variants in the CYP27A1 gene.

The team collected medical history, neurologic and auxiliary examinations, imaging studies, and genetic profiles. The proband underwent whole exome sequencing, and the variants were studied via Sanger sequencing in two affected and five unaffected family members. Two patients in the pedigree exhibited compound heterozygous missense variants in the CYP27A1 gene: c.379C>T, and c.397T>C, both located in exon 2. A literature review focused on China CTX cases, and selecting only studies published in English, revealed that c.1263+1G>A and C.379C>T are the most common variants in genetically diagnosed Chinese CTX patients. Their findings expand the understanding of the genetic and clinical spectrum of CTX and provide significant insights for its diagnosis.

